# Will a Biological Database Be Different from a Biological Journal? 

**DOI:** 10.1371/journal.pcbi.0010034

**Published:** 2005-08-26

**Authors:** Philip Bourne

The differences, or otherwise, between biological databases and journals is an important question to consider as we ponder the future dissemination and impact of science. If databases and journals remain discrete, our methods of assimilating information will change relatively little in the years to come. On the other hand, if databases and journals become more integrated, the way we do science could change significantly. As both Editor-in-Chief of *PLoS Computational Biology* and Codirector of the Protein Data Bank (PDB), one of the oldest and widely used data resources in molecular biology, the question is particularly pertinent. Here, I give my perspective on what could and, I believe, should happen in the future.

My vision is that a traditional biological journal will become just one part of various biological data resources as the scientific knowledge in published papers is stored and used more like a database. Conversely, the scientific literature will seamlessly provide annotation of records in the biological databases. Imagine reading a description of an active site of a biological molecule in a paper, being able to access immediately the atomic coordinates specifically for that active site, and then using a tool to explore the intricate set of hydrogen-bonding interactions described in the paper. Not only are the data generated by the experiment immediately available within the context of what you are reading, but specific tools for interpreting these data are provided by the journal. Alternatively, if you are starting with the data, for example, viewing the chromosome location of a human single-nucleotide polymorphism associated with a neurological disorder, you can immediately access a variety of papers ranked in order of relevance to your profile, not just through links to abstracts but also by pinpointing the reference to the single-nucleotide polymorphism in the full-text article. The type and order of articles displayed could be different, depending on whether you are, for example, a molecular biologist or a neurosurgeon. At this point, whatever your user profile, the distinction between a database and a journal article disappears. How could this happen? To answer this question, we must think about the parallels that exist today between biological databases and biological journals.

The daily work of any high-throughput scientific journal or biological database consists of information input, information processing, and information output. Consider the parallels between a journal and a database for each of these three steps. On a daily basis, the journal accepts manuscripts; once these have been checked for format compliance and completeness, they undergo review, either by an internal group of scientific editors or, as is the case for *PLoS Computational Biology,* through peer review by the scientific community. Likewise, a biological database such as the PDB accepts submissions from the community, which are checked for format compliance and reviewed internally by experienced annotators. There are even parallel presubmission steps in journals and databases. For example, potential authors in *PLoS Computational Biology* may make presubmission inquiries to confirm the suitability of their paper, and depositors to the PDB may run their entries against a validation server to determine whether the data are in compliance, prior to having the same tests run by a PDB annotator.

Once registered with the corresponding online submission system, a journal manuscript receives a permanent manuscript number, while a database entry receives a unique identifier. Subsequent revisions can be mapped to these respective numbers, so that both journals and databases can provide an accurate audit trail of journal manuscripts and database entries, respectively. Once a manuscript or entry is accepted as compliant, both undergo review processes involving one or more iterative steps between institution and author, as the manuscript or the entry is refined and finally released. Release cycles of journals and databases have also become similar—journals such *PLoS Computational Biology* have an option for early online release as soon as the manuscript is accepted, and biological databases typically release entries on a daily or weekly basis, as soon as they have been processed.

Not only are the daily operations of databases and journals similar, but the business models also have parallels (I will not dwell on them here though). Certainly from a consumer's perspective, in terms of accessibility, there is no difference between a paper in a PLoS journal and an entry in the PDB database—they are freely available to all. In the case of open-access journals and open archives like the PDB, the parallels, from the perspective of the consumer, are even more profound than just free access yet are frequently overlooked. PLoS articles are published under a Creative Commons Attribution License, which means that the contents (text and images) of all PLoS journals can be used as the consumer sees fit, provided original attribution is given to the appropriate authors and source. So it is with the contents of many biological databases, including the PDB. Consumers are free to take and analyze the contents by any means they see fit, but are expected to attribute information to the authors of the original material, as appropriate. Finally, in the case of PLoS journals, the copyright of the material is not signed over to the publisher but remains with the original author, which is also true of information provided to most biological databases. In both forms of open access—journals and databases—the only requirement is to provide an immutable reference to the material. In the case of an online journal article, this reference most often takes the form of a digital object identifier (DOI), and for a database entry, it is usually a unique accession number. Like the contents of manuscripts and database entries, I expect these two forms of immutable identifiers to become indistinguishable from each other, as I will outline subsequently.

Given these parallels, at this point in time, what is the difference between an entry in a database and an article in a journal? Currently the difference can be characterized as a mix of perception and content. Clearly, no one perceives a database entry of, say, a sequence, or a specimen in a museum collection, as being as valuable as the journal paper that describes it. But, ironically, to the consumer, at least by one measure, the database entry may indeed be more valuable. The structure of human deoxyhemoglobin is one of the most downloaded structures in the PDB—in one year, it has been downloaded more times than the original paper has ever been cited thus far. Yet from the authors' perspective, the Nobel Prize does not come from constructing the PDB database entry, but from an eloquent description of the relationship between structure and function that was presented most completely in the literature. A tenure committee does not award tenure based on the number of deposits a faculty member has made to a biological database, but rather the number of papers they have published in leading journals.

Those of you who have made it this far might be thinking it is ridiculous that I should regard the content of a database entry in the same way that I regard the content of a scientific paper, given these differences in perception and content. It is possible, though, that you are thinking this way based on traditional perceptions of content and not the way things should be, going forward, given current technologies and social practices. To set the stage for the subsequent discussion, I will highlight three current observations that are relevant to this assertion.

First, publishers have embraced the Internet as a distribution medium but, for the most part, have not used the medium beyond that, simply distributing material in the same way as in printed form. Hyperlinks in documents and citation indexes are exceptions, but compared to what many biological database developers have achieved in terms of information integration and comprehension through novel display techniques, such added functionality is minimal. Second, online journals have greatly reduced the necessity for page limits on papers, since the costs of supporting a long versus short paper are much less online than in the printed form. Journals publishing both online and in print solve this size problem by having short articles in print and placing additional material as supplements in an online form only. This practice has increased dramatically in the past few years: consider the amount of supplementary material in one issue of the *Proceedings of the National Academy of Sciences of the United States of America* today versus five years ago. Supplementary material can be a valuable addition or, alternatively, can make for a disjointed piece of work. Moreover, the supplemental material is ad hoc and cannot be readily queried across all articles, even though a small amount of it is already tagged and comes directly from a database. Third, the perceived value of both a database entry and a journal article has changed over the years. As high-throughput techniques have become more prevalent, data are produced at an ever-increasing rate, so the value of a unit of data, for example, a sequence or structure, has diminished. Data producers hoard their data less than they did in past years. Similarly, the rate of publication has increased dramatically, this increase being brought about by accelerated technologies for manuscript production, large collaborative studies, and increased emphasis on the notion of “publish or perish.” In short, journal content is already becoming more like database content and vice versa.

Can this trend continue? Consider how the respective content of journals and databases is organized. Both have varying degrees of content organization. Papers have structure, but the organization of their content is less detailed than that found in a database, although this is changing with formal document type definitions being applied, from which database schema can be generated. Typically a paper has an introduction, a materials and methods section, a results section, and a discussion section; it possibly uses consistent terms for genes, enzymes, and diseases; and in a post-production step, keywords and/or medical subject headings for indexing the content of the article are added. Databases, on the other hand, frequently have a high level of organization, where data are granular and each granule is described in exquisite detail. The advantage of a paper is that it is relatively easy to input and maintain, but it requires human recall. Machine-based recall of meaningful information is poor, a problem being addressed but certainly not solved by the discipline of natural-language processing. A database, on the other hand, has excellent recall but requires much effort to organize and is best suited to quantitative data, not free text. I would contend that the future offers some middle ground for content organization.

We have taken the first steps toward a middle ground by making both the combined contents of biological databases and biological literature freely available in electronic form. Is the technology available to support the next steps in integration and is the scientific community ready for such a change? I believe that the answer to the technology part of the question is yes. I do not know the answer to the second part, but I think it's time for some preliminary experiments to find out. I would be most interested in hearing views on the matter and any suggestions for potential experiments. In the interim, here are a few experiments I am proposing.

As mentioned above, DOIs provide an immutable reference to a scientific document that exists online. The way I think about DOIs is the same way I think about addresses used to identify computers on the internet, each address possesses a unique identifier that in a seamless way can be resolved to access that specific computer. So it is with DOIs, which can be resolved not only to find the material referenced by the DOI but, through reverse searching, can also be used to find material that references the DOI. Think of what could happen if such DOIs were not only assigned to papers as they are now, but also to items of content within biological databases—protein structures, species distributions, neuroimaging datasets, and so on—and if these DOIs were referenced when that content was used or discussed elsewhere. An immediate outcome would be the ability to find all papers that reference a particular sequence motif, for example: a level of detail that is not currently available to someone accessing a sequence database. Conversely, accessing a paper would immediately provide a resolvable list of the sources of data used in the experiments, which could be accessed and further analyzed—a step toward achieving true reproducibility of an experiment, where the paper has become the interface to the data. Unfortunately, DOIs cost money, and providing a fine level of granularity, such as all sequence motifs for every sequence in the Protein Families Database of Alignments and HMMs, would be prohibitively expensive. Publishers should collaborate with the major database providers, so that database providers provide the appropriate immutable references and published articles reference them.

As another experiment, what if the data in an online paper became more alive? Some databases let you download data into spreadsheets or other client-side applications that render and analyze data. Papers could be treated this way, too. The technology is there to create these ubiquitous clients that are independent of operating systems and hardware and that are downloadable on demand. New levels of comprehension might be achievable. The first step would be to provide tools that better visualize specific types of biological data, without the need for specialized knowledge in using an esoteric tool. Later would come tools for basic analysis, for example, simple statistical tests or principle-component analysis.

Consider one final experiment, what if papers were made to show a higher level of organization than is possible today? Clearly, too much additional work by the author would be resisted, unless it bought clear rewards. Nevertheless, tools can be envisaged that, with minimal work by the author, would further classify the text such that, for example, annotation associated with a particular gene or set of genes is identified, or a set of keywords is generated to be associated with the paper as metadata, and all the author would have to do is confirm their validity. Recent benchmarks indicated that 80% of terms such as gene names could be identified automatically and hence associated with systematic annotation, which could simply be accepted or rejected by the author [1]. Would an author do it, if it led to more rapid citations? I would say so! This type of experiment has already proved to be successful in the community engaged in small-molecule structure determination, although without the data being publicly accessible in an easy way. With the incentive for more citations, the author would review the proposed systematic nomenclature, and we would then have the potential for a new association between the text of a paper and, say, a gene and the description of that gene in a database. If the connection is transparent to the reader, the paper has thus become a detailed entry point to the database and the database has become a detailed entry point to the literature.

These experiments, if successful, would go a long way in answering the question posed here—Is a biological database any different than a biological journal? I am working toward reaching an answer of, no, there is no difference. If you want to help answer this question, I would welcome hearing from you; after all, journals, like databases, should be community resources. 

## Abbreviations

DOI: digital object identifier
PDB: Protein Data Bank
